# Tracing cellular sources of pathogenic type I-interferon in the TREX1^-/-^ mouse model of lupus like-disease

**DOI:** 10.1186/1546-0096-13-S1-O34

**Published:** 2015-09-28

**Authors:** K Peschke, K Frenzel, M Achleitner, M Kleefisch, A Gerbaulet, M Prinz, A Roers, R Behrendt

**Affiliations:** 1 Medizinische Fakultät Carl Gustav Carus TU Dresden, Institute for Immunology, Dresden, Germany; 2 Universitätsklinikum Freiburg, Institute of Neuropathology, Freiburg, Germany

## Introduction

Loss of function mutations of the intracellular enzyme 3’ repair exonuclease (TREX) 1 cause Aicardi-Goutières syndrome (AGS). As AGS clinically overlaps with systemic lupus erythematosus (SLE) and, like SLE, features a spontaneous activation of the antiviral type I-interferon (IFN) system as well as production of antinuclear autoantibodies, this condition may be considered a monogenic variant of SLE. TREX1-deficient mice spontaneously develop multiorgan autoimmune disease that is fully dependent on a functional type I-IFN system. This phenotype suggested a new concept of systemic autoimmunity arising from intracellular accumulations of (so far enigmatic) nucleic acid substrates of TREX1, which trigger chronic antiviral IFN responses and thereby autoimmunity. Nonhematopoietic cells were proposed to be the cellular source of the pathogenic IFN. Uncontrolled activity of endogenous retroelements was suspected to induce the chronic antiviral response.

## Objectives

We sought to identify cell types responsible for the loss of self tolerance and to clarify whether there is a role for endogenous retroelements in the disease of TREX1-deficient mice.

## Materials and methods

In order to elucidate the role of Trex1 in the hematopoietic system, lethally irradiated mice were reconstituted with TREX^-/-^ fetal liver cells. Furthermore we generated a Trex1 flox mouse line which was used to specifically delete TREX1 in CD11c-expressing cells (CD11c-Cre), in DCs (Clec9a-Cre), and in B cells (CD19-Cre). To test a potential role of retroelements, TREX1^-/-^ were crossed to transgenic retrotransposition reporter mice and were treated with reverse transcriptase inhibitors.

## Results

While Cre/loxP-mediated conditional inactivation of *Trex1* in various non-hematopoietic compartments did not result in detectable inflammatory disease, selective knock out in the hematopoietic system largely reproduced the TREX1^-/-^ phenotype. Mice with inactivation of the *Trex1* gene in DCs developed only mild autoimmunity, but featured massive upregulation of interferon stimulated genes suggesting a critical pathogenic role of DCs. Interestingly, selective loss of TREX1 in CD19+ B cells did not result in a detectable activation of the type I-interferon system.

Treatment of TREX1^-/-^ mice with the reverse transcriptase inhibitor Truvada^®^ did not ameliorate the phenotype. Retrotransposition events were not increased in frequency in a TREX1^-/-^ compared to a wildtype background.

## Conclusion

We demonstrate that specific cell types differentially respond to the loss of TREX1 and show that DCs are an important source of type I IFN in the TREX1^-/-^ model. Our findings that loss of TREX1 had no impact on Line1 retrotransposition and that pharmacological inhibition of retrotransposition in vivo did not rescue TREX1^-/-^mice from lethality challenges the concept of a pathogenic role of endogenous retroelements in this model.

